# Heat sink effect of underwater polypectomy in a porcine colon model

**DOI:** 10.1186/s12876-021-01985-1

**Published:** 2021-10-27

**Authors:** Chih-Wei Tseng, Yu-Hsi Hsieh, Chung-Chih Lin, Malcolm Koo, Felix W. Leung

**Affiliations:** 1 Division of Gastroenterology, Department of Medicine, Dalin Tzu Chi Hospital, Buddhist Tzu Chi Medical Foundation, 2 Minsheng Road, Dalin, Chiayi, 62247 Taiwan; 2 School of Medicine, Buddhist Tzu Chi University, Hualien, Taiwan; 3 Department of Mechanical and Computer-Aided Engineering, National Formosa University, Yilan, Taiwan; 4 Graduate Institute of Long-Term Care, Tzu Chi University of Science and Technology, Hualien, Taiwan; 5 Dalla Lana School of Public Health, University of Toronto, Ontario, ON Canada; 6 Sepulveda Ambulatory Care Center, Veterans Affairs Greater Los Angeles Healthcare System, North Hill, CA USA; 7 David Geffen School of Medicine at UCLA, Los Angeles, CA USA

**Keywords:** Polypectomy, Underwater, Colonoscopy, Heat sink, Temperature

## Abstract

**Background:**

Underwater polypectomy without the need for submucosal injection has been reported. A heat-sink effect by immersing the polyp in water was proposed but no such experiment has been performed to support the claim. We compared the temperature rise on the serosal side during polypectomy between air- and water-filled colon.

**Method:**

Freshly harvested porcine colons were placed in a metal tray with cautery electrode pad attached to its bottom. An upper endoscope was used with a cap and a rubber band mounted to the distal end. A mucosal site was randomly selected and identified on its serosal surface with a marker while suction was applied. Suction was applied again and a ligation band was applied to create a polyp. A cautery snare grasped the artificial polyp just below the band. An assistant placed the tip of a thermometer at the marked site on the serosal surface to record the baseline temperature before cautery and the highest temperature during polypectomy. Seven polypectomies in air and underwater were performed.

**Results:**

Mean (standard deviation) baseline temperature were 23.3 (0.6) °C and 23.4 (0.6) °C in the air and water groups, respectively. The maximum rise in temperature during polypectomy was 6.1 (4.5) °C and 1.4 (1.0) °C in the air and water groups, respectively (*P* = 0.004).

**Conclusions:**

The maximum temperature rise during polypectomy was significantly less when polypectomy was performed underwater, supporting the hypothesis that a heat-sink effect does exist during underwater polypectomy.

## Introduction

Endoscopic resection of colon polyps is estimated to prevent around 80% of colorectal cancers [1, 2]. Early endoscopic mucosal resection (EMR) without submucosal injection had acute and delayed complication, possibly related to “overheating” [3]. Injection of saline solution into submucosal space to lift the lesion away from the muscularis propria was proposed to minimize these complications by reducing the extent of thermal injury [4–6]. However, submucosal injection adds to the procedure time, and sometimes makes the capturing of flat polyps with a snare more difficult [7]. Recently, underwater polypectomy (UWP), which can obviate the need for submucosal injection, was proposed. It has been shown to successfully resect, either by piecemeal or en-block, large (≧ 2 cm) and small polyps, recurrent adenomas following partial resection, and adenomas involving the appendiceal orifice, which are deemed high risk for EMR [8–12]. UWP achieved faster resection for large lesions (> 10 mm) compared to the traditional EMR through avoidance of submucosal injection [13]. A recent meta-analysis also showed UWP was associated with less overall complications (relative risk [RR] 0.66 (95% confidence interval [CI] 0.48–0.90) (*P* = 0.008), and less intra-procedural bleeding (RR 0.59, 95% CI 0.41–0.84, *P* = 0.004) [14].

Several mechanisms have been proposed to explain why UWP could eliminate the need for submucosal injection. As observed with endoscopic sonography, the colonic folds consisted of involutions of the mucosa and submucosa “float up” in water, while the muscularis propria remains circular and does not follow the involutions, so the muscularis propria would unlikely be captured by the snare underwater [8]. Another possible mechanism is the heat-sink effect of water that offers protection against deeper thermal injury during UWP. However, no evidence, clinical or experimental, has yet been presented to substantiate the claim [8]. Therefore, we designed an in vitro experiment to compare the temperature rise on the serosal side of porcine colon opposite to an artificially created polyp undergoing polypectomy either underwater or in an air-filled colon. Our hypothesis is that the temperature rise would be significantly less during UWP compared with polypectomy in an air-filled colon because of a heat-sink effect.

## Methods

The endoscopic procedures were conducted at the endoscopic unit at Dalin Tzu Chi Hospital, Tzu Chi Medical Foundation, Chiayi, Taiwan. The Institutional Review Board approval and informed consent were waived because this was an ex vivo experiment on porcine colon without involving any human organ or live animal.

## Preparation of the porcine colon

Freshly harvested porcine colons were brought to the endoscopy suite from the local market and stored in cool saline until preparation. They were irrigated with copious amount of tap water until the colon lumens were clean. Only the distal 50–60 cm of the colon with intact rectum and anus was used for intubation with the endoscope. The colon was placed in a metal tray, with a cautery electrode pad attached to its bottom. The anus was fixed to the tray with Kelly forceps to facilitate the intubation. The proximal end of the colon was closed with Kelly forceps for a good air-tight effect.

## Creation of artificial polyps

An upper endoscope was used with a cap and a rubber band mounted at the distal end. A mucosal site in the sigmoid colon was randomly selected. First, gentle suction was applied with the scope firmly placed on the mucosal surface to create a dimple at the opposite serosal surface. An assistant located and labeled the site with a marking pen, where a thermometer would be applied to measure the temperature during polypectomy. The suction was released to avoid prolonged suction and subsequent injury to the colon wall. Suction was applied again at the same site at a pressure of − 10 mmHg to create an artificial polyp by using a pneumatically-activated esophageal variceal ligation device (MD-48,709; Sumitoma Corp, Tokyo, Japan) [15]. The serosal surface opposite to the polyp was inspected to make sure that the serosa was not sucked into the polyp. We created 14 artificial polyps in two colons from two pigs.

## Polypectomy and measurement of temperature rise

**Fig. 1:**
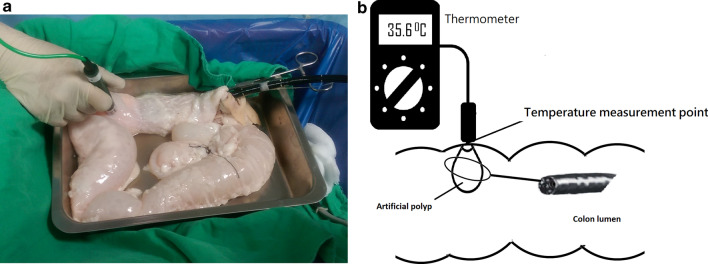
An assistant placed the tip of a thermometer at the marking on the serosal surface opposite to mucosal site of the polyp while polypectomy was being performed

**Fig. 2:**
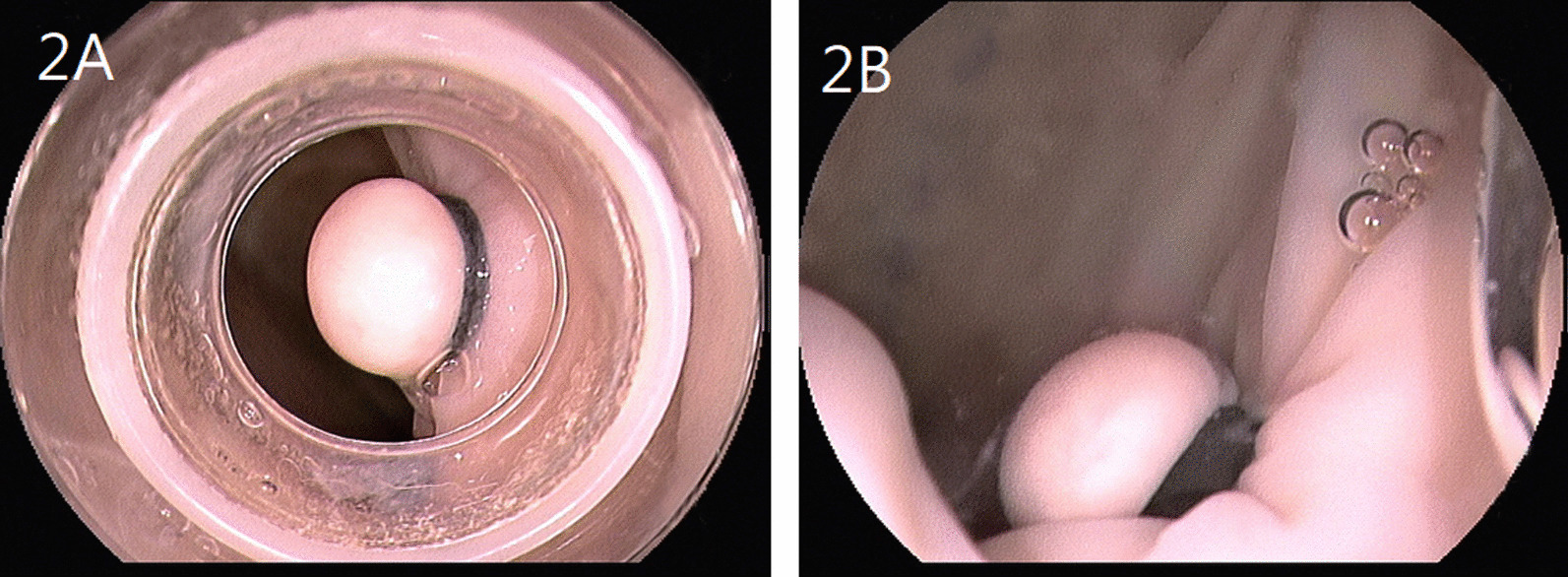
Appearance of artificial polyps created by variceal ligation device. **A** in air, **B** in water

A snare (SD-12U-1, 15 mm, Olympus) was used to grasp the polyp just below the rubber band. An assistant placed the tip of a thermometer (TM-160 A K type, sensitivity 0.1 °C, Horng-Chi, Taiwan) at the marking on the serosal surface opposite to mucosal site of the polyp. (Fig. 1) Baseline temperature was recorded before the cautery was applied. The highest temperature achieved was recorded while the cautery was applied. Equal numbers of artificial polyps (n = 7 in each group) were randomized to undergo polypectomy either in air-filled or water-filled lumen. Room air was insufflated during polypectomy in the air group (Fig. 2A) while water at room temperature was infused to distend the lumen after air was sucked out before polypectomy in the water group (Fig. 2B). The same cautery setting with blended current (ENDOCUT Q, effect 3, 35 W; Erbe) was used in both groups.

### Statistical analysis

The primary outcome was the maximum rise of temperature during polypectomy. Statistical analysis was performed using SPSS version 19.0 software (SPSS Inc., Chicago, Illinois, USA). The data were represented by using mean (standard deviation [SD]). The Mann-Whitney *U* test was used to compare differences in continuous variables between the two groups. A *P* value < 0.05 was considered significant.

## Results

**Fig. 3:**
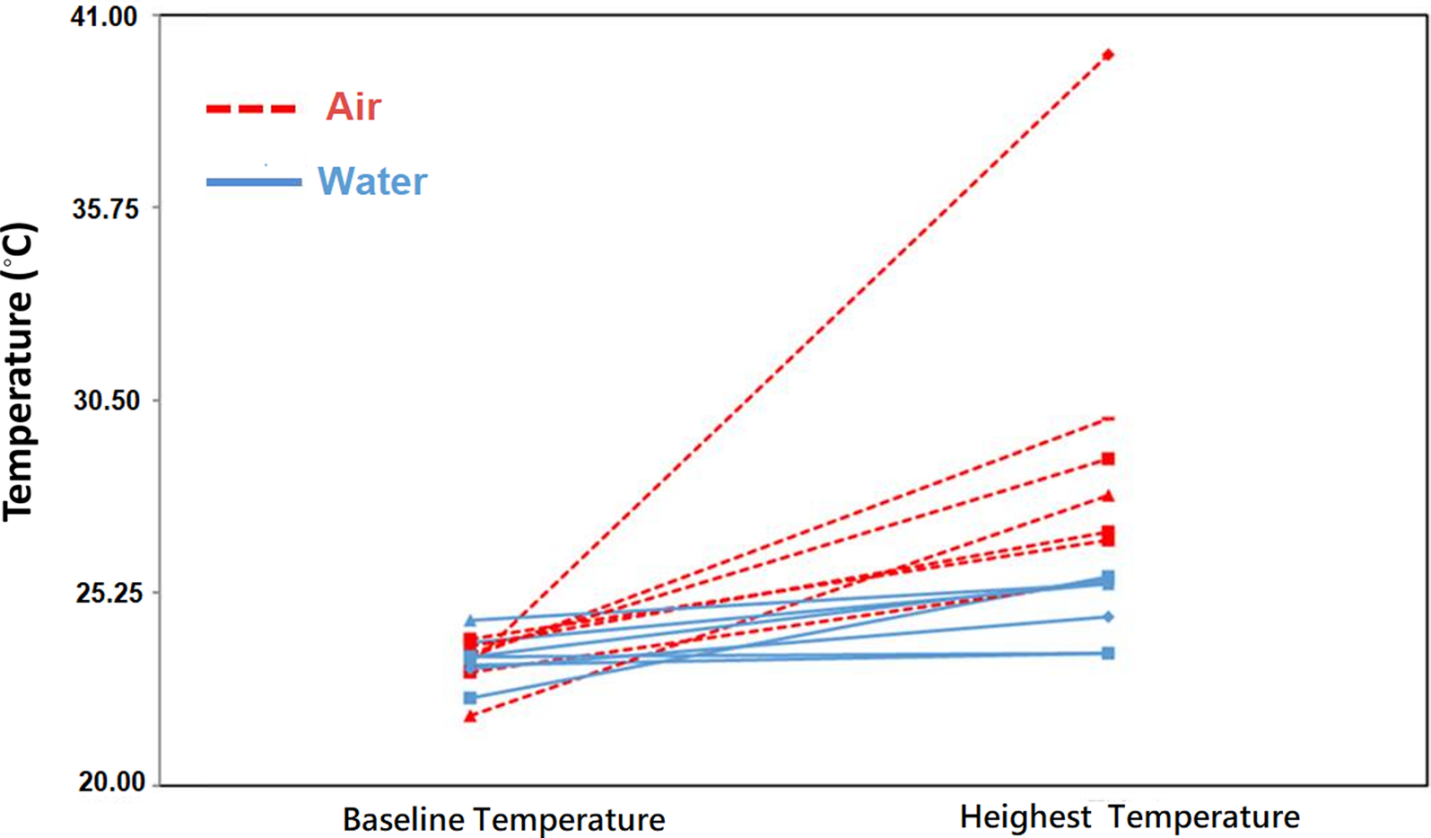
Baseline temperatures and highest temperatures measured on the serosal surface during polypectomy in air (red) and in water (blue)

**Fig. 4:**
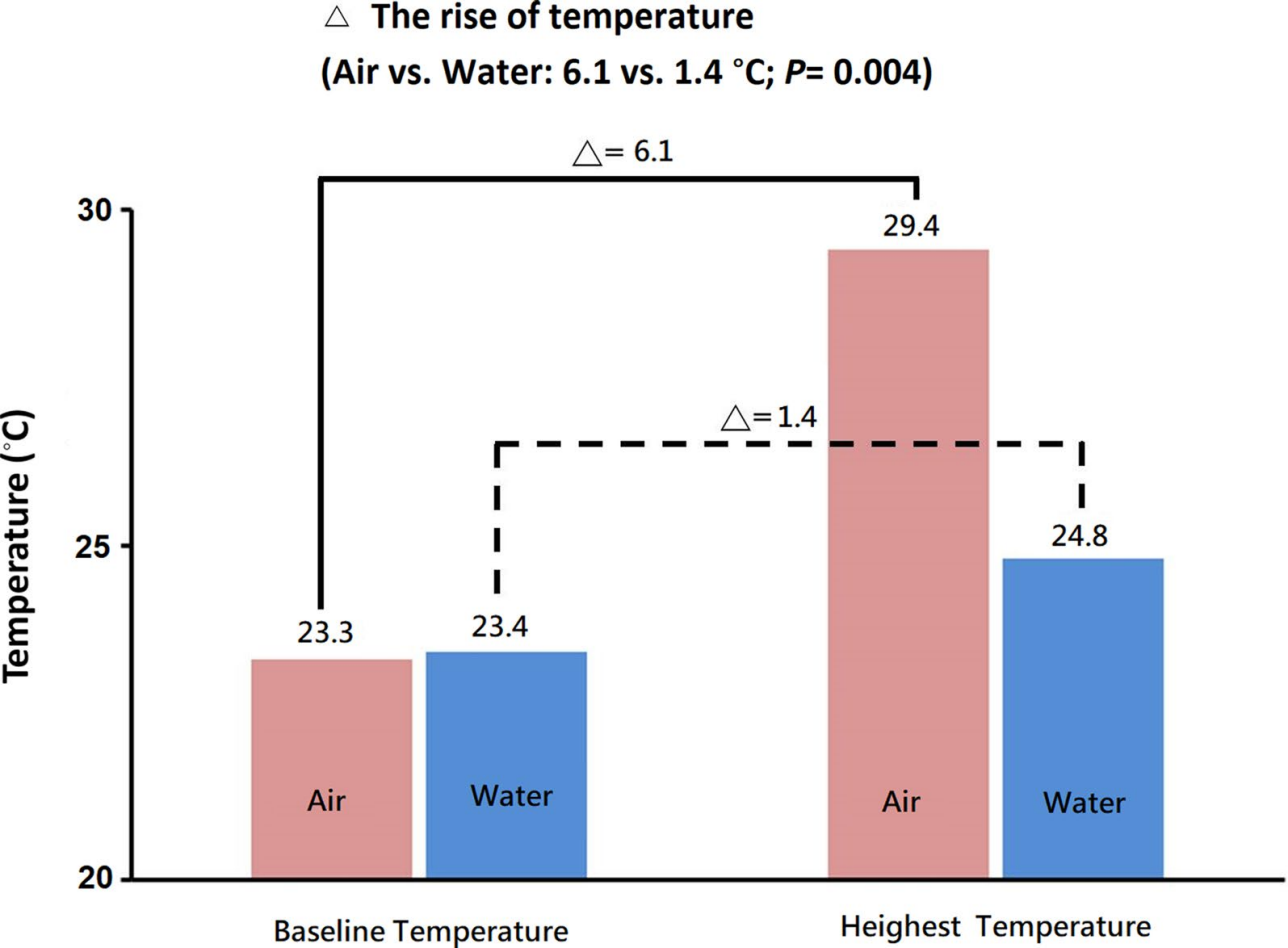
The mean baseline temperature and highest temperature in the air (color red, n = 7) and water (color blue, n = 7) groups. The maximum rise in temperature (°C) during polypectomy was 6.1 (4.5) and 1.4 (1.0) in the air and water groups, respectively (*p* = 0.004)

A total 14 artificial colon polyps were created by rubber band ligation and randomized to undergo polypectomy either underwater (n = 7) or in air-filled lumen (n = 7). The dynamic change of the temperatures during polypectomy were showed in Figs. 3 and 4. The mean (SD) baseline temperatures were similar in both groups (air vs. water, 23.3 [0.6] °C vs. 23.4 [0.6] °C, *P =* 0.902). The mean (SD) highest temperatures (°C) achieved during polypectomy in air group were significantly higher than that in the water groups (29.4 [4.8] °C vs. 24.8 [0.0] °C, *P* = 0.001). The rise in temperature (°C) during polypectomy were significantly higher in the air than that in the water groups (6.1 [4.5] °C vs. 1.4 [1.0] °C, *P* = 0.004).

## Discussion

This ex vivo study demonstrated that the rise in temperature during polypectomy was significantly less when performed underwater than in air-filled lumen, supporting the presence of the heat-sink effect.

While generally considered to be effective and safe, endoscopic polypectomy does carry risks, including postpolypectomy bleeding (0.1–0.6%) [16], transmural thermal injury, and perforation (0.07%) [17].

One recent meta-analysis showed UWP was associated with a significantly lower rate of immediate bleeding compared with conventional EMR. The authors suggested that obviating the need for needle puncture during submucosal injection itself might help reduce the bleeding [14], although less thermal injury to submucosal vessels also might be beneficial.

Delayed postpolypectomy bleeding (DPPB) is associated with thermal injury. Horiuchi et al. compared cold snare polypectomy without electric cautery and conventional hot snare polypectomy with cautery for small colon polyps in anticoagulated patients. No delayed bleeding occurred in the cold snare group, whereas 5 patients (14%) developed DPPB and required endoscopic hemostasis in the conventional group (*P* = 0.027) [18]. The presence of histologically demonstrated injured arteries in the submucosal layer with cold snare was significantly less frequent than those with conventional hot snare (22% vs. 39%, *P* = 0.023), presumably as a result of a lack of thermal injury [18]. In the current study, less temperature rise in the UWP group suggested that there would be less thermal injury to the deeper layers of colon wall. Previous reports showed the rates of DPPB following UWP without submucosal injection for large polyps (≥ 2 cm) ranged from 2 to 5% [4, 6], which did not significantly differ from that of conventional EMR with submucosal injection at rates ranging from 4 to 7% [19, 20]. Recent meta-analyses also showed comparable delayed bleeding rate between UWP and EMR [14]. It appears that UWP has a similar protective effect to submucosal injection as far as DPPB is concerned.

In addition, electrocautery associated thermal injury could result in post polypectomy syndrome and was reported to account for 18% of colon perforation [17]. Initial reports on UWP for polyps ≥ 2 cm showed no perforation or transmural thermal injury, attesting to the protective effect of water. A perforation case following UWP was subsequently reported [21]. The authors attributed the perforation in the ascending colon to stretching of the colon by the retroflexed scope, preventing the lesion from “floating.” Recent meta-analyses comparing UWP with conventional EMR showed similar safety profile in terms of transmural thermal injury and perforation, attesting to the presence of heat sink effect [14, 22, 23].

There are several limitations in our study. First, a porcine colon was used instead of a human colon in this study. Second, a lack of blood flow in the ex vivo colon model might affect the results. Third, polypectomy was performed on artificial polyps created by using band ligation device instead of real polyps. Nevertheless, the study also has some strengths. The use of the ex vivo colon model allowed us to directly measure the temperature on the colon wall, which would be very difficult to achieve when polypectomy is actually being performed in the human body. Performing studies in living pigs would be a reasonable next step.

In addition to measuring the temperature change on the serosal surface of the polypectomy site, future studies should also measure the temperature change in the water and the intensity of the temperature change, i.e., the product of the degree and duration of temperature change. Evaluating the extent of the thermal injury caused by the cautery by examining the histology of the respected polyps and corresponding wounds on the colon wall would be informative. It might also be interesting to evaluate the heat-sink effect on polyps of larger size, since larger polyps usually carry higher risk of complications of polypectomy.

## Conclusions

In conclusion, the maximum temperature rise during polypectomy was significantly less when polypectomy was performed underwater in a porcine model of polypectomy. These data support the presence of a heat-sink effect during UWP.

## Data Availability

Data sharing is not applicable to this article as no datasets were generated or analysed during the current study.

## Abbreviations

CI: Confidence interval
DPPB: Delayed postpolypectomy bleeding
EMR: Endoscopic mucosal resection
RR: Relative risk
SD: Standard deviation
UWP: Underwater polypectomy
